# Examining Patient Engagement in Chatbot Development Approaches for Healthy Lifestyle and Mental Wellness Interventions: Scoping Review

**DOI:** 10.2196/45772

**Published:** 2023-05-22

**Authors:** Chikku Sadasivan, Christofer Cruz, Naomi Dolgoy, Ashley Hyde, Sandra Campbell, Margaret McNeely, Eleni Stroulia, Puneeta Tandon

**Affiliations:** 1 Department of Medicine University of Alberta Edmonton, AB Canada; 2 Department of Physical Therapy University of Alberta Edmonton, AB Canada; 3 John W Scott Health Sciences Library University of Alberta Edmonton, AB Canada; 4 Department of Computing Science University of Alberta Edmonton, AB Canada

**Keywords:** chatbots, virtual assistants, patient involvement, patient engagement, codevelopment

## Abstract

**Background:**

Chatbots are growing in popularity as they offer a range of potential benefits to end users and service providers.

**Objective:**

Our scoping review aimed to explore studies that used 2-way chatbots to support healthy eating, physical activity, and mental wellness interventions. Our objectives were to report the nontechnical (eg, unrelated to software development) approaches for chatbot development and to examine the level of patient engagement in these reported approaches.

**Methods:**

Our team conducted a scoping review following the framework proposed by Arksey and O’Malley. Nine electronic databases were searched in July 2022. Studies were selected based on our inclusion and exclusion criteria. Data were then extracted and patient involvement was assessed.

**Results:**

16 studies were included in this review. We report several approaches to chatbot development, assess patient involvement where possible, and reveal the limited detail available on reporting of patient involvement in the chatbot implementation process. The reported approaches for development included: collaboration with knowledge experts, co-design workshops, patient interviews, prototype testing, the Wizard of Oz (WoZ) procedure, and literature review. Reporting of patient involvement in development was limited; only 3 of the 16 included studies contained sufficient information to evaluate patient engagement using the Guidance for Reporting Involvement of Patients and Public (GRIPP2).

**Conclusions:**

The approaches reported in this review and the identified limitations can guide the inclusion of patient engagement and the improved documentation of engagement in the chatbot development process for future health care research. Given the importance of end user involvement in chatbot development, we hope that future research will more systematically report on chatbot development and more consistently and actively engage patients in the codevelopment process.

## Introduction

Growing evidence supports the use of digital technology in healthy eating, physical activity, and mental wellness interventions. Several systematic reviews on these digital health interventions (DHIs) have identified their promise in managing chronic diseases [1-6]. Specifically, DHIs have proven impacts on reducing risk factors for chronic diseases [3,4] by increasing physical activity, reducing body mass index [6], and improving patient psychosocial well-being [2]. Further, DHIs can help overcome barriers to access to mental health support for individuals with chronic conditions [1]. Although these DHIs are useful in vulnerable chronic disease populations [5,7], they face challenges, including limited user adoption, low engagement, and high attrition rates [8-11].

Chatbots are artificial intelligence (AI) programs that converse with humans through natural language in text or speech [12]. There is a growing body of evidence that the integration of chatbots into DHIs may provide support [13-17] by increasing patient engagement [13], intervention adherence [13], and the acceptability and efficacy of lifestyle and wellness interventions [15-17]. Additionally, chatbots offer a range of potential benefits to end users and service providers, most notably allowing for more scalable, cost-efficient, and interactive solutions [12].

Although developments in AI and computer science have improved the ability of chatbots to mimic human agents, the acquisition of a relevant data set with which to train chatbots remains challenging. User-centered design with public and patient involvement (PPI) may offer a potential solution [18-20]. By engaging key stakeholders, PPI can help produce better-quality interventions relevant to end users’s needs [18], resulting in benefits such as increasing intervention acceptability, effectiveness, and sustainability [19]. Drawing on evidence across other digital health care innovations, the proposed benefits of PPI fundamentally include the development of interventions that are both usable by and relevant to patients [19]. Recognizing the limited data available to guide the role of PPI in digital health innovation, experts have called for the meaningful involvement of patients from the beginning of the development process to allow for the cocreation of relevant, valuable, and acceptable digital health solutions [20].

This scoping review aimed to map the literature on studies using chatbots to engage in 2-way natural language interaction (voice- or text-based input) to aid the delivery of healthy eating, physical activity, and mental wellness interventions. The specific objectives of this review were: (1) to report the nontechnical (eg, unrelated to software development) approaches for chatbot creation and (2) to examine the level of patient engagement in these reported approaches. Although the technical software development steps are essential to creating chatbots, this review focused on the nontechnical approaches for chatbot development as these are less explored and more likely to involve patient participation. To our knowledge, this is the first scoping review to systematically explore these objectives.

## Methods

### Study Design

This scoping review was conducted using the framework proposed by Arksey and O’Malley [21] and later refined by Levac et al [22]. The Arksey and O’Malley framework consists of the following five steps: (1) identify a research question, (2) identify relevant studies, (3) select studies, (4) chart the data, and (5) summarize and report the results [21]. Two research questions guided the review:

1. Outside of the technical software development processes, what approaches are described for the development of chatbots that support healthy eating, physical activity, and mental wellness interventions?
2. What is the extent of patient engagement in these approaches?

### Study Team

Our multidisciplinary study team included 2 graduate student researchers (CS and CC), a health sciences librarian (SC), 2 postdoctoral fellows with backgrounds in clinical care and scoping reviews (ND and AH), a professor of medicine (PT), a professor of physiotherapy (MM), and a professor of computing science (ES).

### Search Strategy

A health sciences research librarian (SC) was consulted to develop a search strategy that used concepts from our research questions. The search strategy (Textbox 1) included a combination of subject headings and keywords, including health, chatbots, and lifestyle or wellness components. Searches were adjusted appropriately for each database. Nine electronic databases were searched in July 2022 including OVID MEDLINE, OVID Embase, OVID PsycINFO, EBSCO CINAHL, Scopus, IEEE Explore, Proquest Dissertations and Theses Full Text, Cochrane Library, and PROSPERO (International Prospective Register of Systematic Reviews). No publication date limit was applied to the search, as the literature on chatbots and virtual conversation agents is naturally self-limiting. After conducting the search, the results were imported into Covidence systematic review management software and duplicates were removed [23]. Covidence is a “web-based collaboration software platform that streamlines the production of systematic and other literature reviews” [23]. The full text of the search strategy is in Multimedia Appendix 1.

**Search strategy used for OVID PsycINFO database.**

# Searches

1. (chatbot* or “im bot” or “im bots” or “instant message bot*” or “conversational agent*” or “virtual agent*”).mp.
*“Diets”/
*“Health Promotion”/
*“Intervention”/
*“Physical Activity”/
6. “Nutrition”!
7. “Weight Loss”!
8. “Sedentary Behavior”/
9. (lifestyle* or health* or medic* or nursing or nurse* or disabilit* or elder* or “senior citizen*” or patient* or exercise or “physical activit*” or motivational).mp.
10. 2 or 3 or 4 or 5 or 6 or 7 or 8 or 9
11. 1 and 10

### Eligibility Criteria

Included publications were those written in English and published in peer-reviewed journals. Included studies all had an intervention supporting healthy eating, physical activity, and mental well-being. All studies required a chatbot that communicated with users through a 2-way natural language interaction. Inclusion criteria for participants consisted of adolescents (age >10 years old) as defined by the World Health Organization [24] or adult populations. Studies were excluded if they involved additional technologies or chatbot service delivery beyond the scope of this review (ie, embodied conversation agents, humanoid and social robots, wearable technology, Internet of Things (IoT), virtual avatars, interactive voice assistants, or chatbots delivering therapy to clients). Studies were also excluded if they only described an intervention but did not conduct or test one. Chatbots designed to replace a therapist’s role were excluded, as were papers that did not present original results (ie, reviews and protocol papers). Randomized controlled trials (RCTs) were included in recognition that they often contain valuable insights into the development process, particularly when the authors did not publish a formative manuscript.

### Study Selection

Titles and abstracts of the retrieved articles were reviewed independently by 2 researchers (CS and CC) based on the inclusion and exclusion criteria described above. Both reviewers met throughout the title and abstract screening stage to discuss and resolve conflicts through consensus. A third reviewer (ND or AH) was consulted for consensus. The remaining articles advanced to the full-text screening stage. The excluded articles were tagged with reasons for exclusion derived from our exclusion criteria. After independent full-text screening, both reviewers met to resolve any inclusion or exclusion and exclusion reason conflicts. Interrater reliability was assessed using the Cohen κ [25]. For the included articles, an additional literature search was carried out using the involved authors, chatbot details, and reference lists to determine whether the previous formative papers that described the chatbot development had been published.

### Data Extraction

One reviewer (CS) extracted the data from included articles using a standardized Microsoft Excel form. General and specific data were extracted, including author, publication year, journal, study setting, study design, sample size, participant demographics (age, sex, and chronic disease where applicable), intervention type, chatbot type, chatbot development approaches, and assessment of patient involvement in development.

Patient involvement was assessed using the Guidance for Reporting Involvement of Patients and Public (GRIPP2) short-form checklist [26]. The GRIPP2 checklist was applicable for our objectives as it was designed to enhance the quality of patient and public involvement (PPI) reporting in health technology assessment and health research [26], and because it could be used retrospectively to measure the quality of PPI reporting in publications and reports [27]. Table 1 depicts the GRIPP2 checklist as we used it to assess PPI in chatbot development. The GRIPP2 awards points across 5 items that describe public engagement and involvement.

**Table 1:** How the Guidance for Reporting Involvement of Patients and Public (GRIPP2) reporting checklist was used to grade patient and public involvement in chatbot nontechnical development.^a^

Section and topic	Specifics for engagement in chatbot-related development
1. Aim	Report the aim of PPI^b^ in chatbot development
2. Methods	Provide a clear description of the methods used for PPI in chatbot development
3. Study results	Outcomes: Report the results of PPI in chatbot development, including both positive and negative outcomes
4. Discussion and conclusions	Outcomes: Comment on the extent to which PPI influenced chatbot development overall. Describe positive and negative effects
5. Reflections or critical perspective	Comment critically on chatbot development, reflecting on the things that went well and those that did not, so others can learn from this experience

^a^Adapted from Staniszewska et al [27].

^b^PPI: patient and public involvement.

## Results

### Search Results

**Figure 1:**
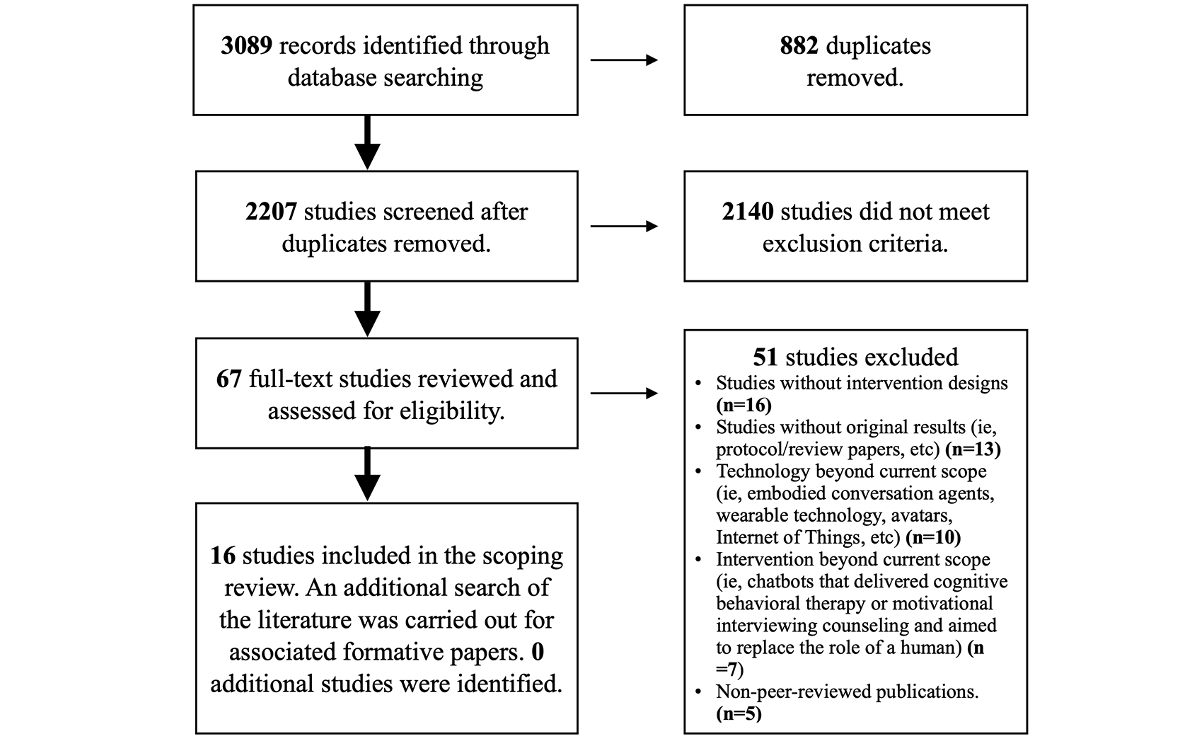
PRISMA (Preferred Reporting Items for Systematic reviews and Meta-Analyses) flow diagram of included and excluded studies.

Figure 1 shows the search results; 3089 publications were retrieved from the database searches, and 882 duplicates were removed, leaving 2207 studies to screen. At the title and abstract screening stage, there was “fair” agreement between reviewers (Cohen κ=0.309, proportionate agreement=0.967). After completing the title and abstract screening, 2140 publications were removed as they did not meet the inclusion criteria. Reading the full text of the remaining 67 publications resulted in a further 51 publications being excluded, with the exclusion reasons documented in Figure 1. At the full-text review stage, there was “almost perfect” agreement (Cohen κ=0.843, proportionate agreement=0.941). In total, 16 publications were included in this review.

### Description of Included Studies

Table 2 shows the description of the included studies and their chatbot interventions. The included studies were conducted in 4 countries, with 50% (8/16) of the studies conducted in Canada [28-35]. Six studies were conducted in Switzerland [36-41], 1 study was conducted in Saudi Arabia [42], and 1 study was conducted in Korea [43]. The majority of the studies (14/16) were conducted in a health care setting [28-40,43], with the remaining 2 studies in a computing science setting [41,42]. All but one of the included studies [31] were published in 2020 or later.

**Table 2:** Descriptive summary of included studies, chatbots, and their development.

Study and country	Study type	Chatbot intervention	Approaches for development	Identified development approaches	Patient engagement (GRIPP2^a^)
Alghamdi et al [42], Saudi Arabia	Randomized controlled trial	Text-based nutrition chatbot for patients with celiac disease	Literature review of existing health behavior change models. Investigated the pros and cons of each model to guide development of a health behavior change model to structure the chatbot's content Interviews with expert users (from patient population diagnosed with celiac disease 4+ years ago, patient’s parent, dietitian supervising patient for 4+ years, gastroenterologist treating celiac disease patient for 4+ years) Questionnaires for patients with celiac disease to understand symptoms and technology use preferences	Literature review Patient interviews Collaboration with knowledge experts	Unable to assess
Davis et al [36], Switzerland	Nonrandomized experimental study	Text-based exercise and nutrition chatbot	Development outsourced to a software company; did not report any steps taken for development	None identified	Unable to assess
Dhinagaran et al [28], Canada	Feasibility study	Text-based exercise, nutrition, and wellness chatbot for patients with diabetes	Needs assessment conducted in an earlier publication Literature review of systematic reviews and clinical guidelines for evidence-based content development to develop content After a 4-week pilot feasibility study, conducted follow-up interviews to understand patient views of the chatbot and to gain ideas for improvement	Literature review Patient interviews	Unable to assess
Figueroa et al [37], Switzerland	User design study	Text-based exercise chatbot	Qualitative interviews during prototype testing to assess opinions and knowledge of chatbots as personal health coaches, technology use, digital literacy, and privacy considerations of chatbots in general Wizard of Oz procedure. Participants completed a 20-minute SMS text messaging conversation with a simulated chatbot Chatbot prototype testing. Participants texted the prototype for 10-20 minutes. Directly after the testing period, participants had a semistructured interview via videoconference regarding the chatbot’s ease of use, usefulness, humanness, and sustainability Co-design workshop for participants to take part in development of ideas for chatbot use and design. These workshops were held over Zoom and ideas were visualized on Google Jamboard	Patient interviews Wizard of Oz procedure Prototype testing Co-design workshops	Met criteria on GRIPP2 checklist points 2, 4, and 5. Provided a clear description of the methods used for PPI^b^, commented on how PPI influenced the study, and on successful and unsuccessful aspects of the study relating to PPI
Gabrielli et al [29], Canada	Proof-of-concept study, mixed methods	Text-based wellness chatbot	Intervention design. The intervention, targets, and components were defined to specify clinically relevant effects on users and to refine the intervention components. This was done by a team of 3 clinical psychologists, 2 users, and behavior change experts Preliminary testing. A proof-of-concept implementation of the digital intervention and chatbot to examine engagement and effectiveness with a convenience sample of university students	Collaboration with knowledge experts Prototype testing	Unable to assess
Gabrielli et al [30], Canada	Pilot, co-design study	Text-based wellness chatbot	Co-design workshop. The students used and commented on a prototyped session of the chatbot intervention to collect their needs and preferences on the following: the chatbot’s look and feel, the type of content and duration of the session, their unmet expectations regarding the prototype, and suggested improvements Feasibility test. This formative study aimed to assess the perceived value of the coaching intervention and to check the user experience with intervention to refine content	Co-design workshops	Met criteria on GRIPP2 checklist point 2. Provided a clear description of the methods used for PPI
Greer et al [31], Canada	Randomized controlled trial	Text-based wellness chatbot for patients with cancer	Literature review of the Stress and Coping theory and the Broaden-and-Build theory of positive emotion and focused on the teaching and practice of 8 positive psychological skills. Created lessons based on this review for the chatbot to deliver Interviews and focus groups as formative work to refine content for the chatbot format and inform adaptation for delivery to a young user base with a shared experience of cancer treatment	Literature review Patient interviews	Unable to assess
Issom et al [38], Switzerland	Usability study	Text-based exercise, nutrition, and wellness chatbot for patients with SCD^c^	Literature review of evidence-based knowledge of SCD self-management, in addition to consulting the World Health Organization’s handbooks on how to implement text-based mHealth interventions to help with dialogue design	Literature review	Unable to assess
Krishnakumar et al [32], Canada	Nonrandomized experimental study	Text-based exercise and nutrition chatbot for patients with type 2 diabetes mellitus	Literature review to develop a lesson plan of the program. This was based on the American Association of Diabetes Educators’s AADE7 self-care behaviors	Literature review	Unable to assess
Larbi et al [39], Switzerland	Usability study	Text-based exercise chatbot	Literature review of behavior change interventions Summarized and briefly reported 4 steps in development: strategy planning, design, implementation, and testing. As part of strategy planning, psychology and public health experts were interviewed Also stated that the development of the prototype involved 3 steps: requirement analysis, concept development, and implementation. Reporting did not go into any further detail	Literature review	Unable to assess
Maenhout et al [40], Switzerland	Development pilot study	Text-based exercise, nutrition, and wellness chatbot	Intervention planning through a scoping review of literature, conducting focus groups, and consulting web-based chat threads for a youth helpline. Focus groups addressed: content preferences, design preferences, questions that the chatbot would be asked, and answers that were expected from the chatbot Intervention optimization through conducting a log data analysis during pretesting. A prototype of the chatbot was developed and pretested by the target users. The prototype was developed based upon guidance from phase 1 focus groups. Conversation logs were closely monitored to refine and fine-tune the chatbot. A question list was formed at the end of this prototype testing phase, 37 new (and practical) questions originated that were not covered in the chat threads and focus groups	Literature review Patient interviews Prototype testing	Met criteria on GRIPP2 checklist point 2
Maher et al [33], Canada	Proof-of-concept study	Text-based exercise and nutrition chatbot	Did not report how the chatbot was developed; the methods section described how the pilot study was conducted	None identified	Unable to assess
Pecune et al [41], Switzerland	Nonrandomized experimental study	Text-based nutrition chatbot	Literature review of persuasive systems, recommender systems, and food-related experiments Collected a food database by regrouping the 40 ingredients that people most frequently cook and eat for dinner. These data were collected from hundreds of participants through questionnaires Completed a pilot study to determine what the critical elements are for recipe recommendation systems. Also, completed this quasi-experimental study to understand the efficacy of different chatbot characteristics with the target end user group	Literature review	Unable to assess
Piao et al [43], Korea	Usability study	Text-based exercise chatbot	Needs assessment through web-based surveys to assess daily routines of office workers (the target group). This was used to determine daily activities that were measurable and easy to execute. These became a part of the goal setting in the intervention Chatbot design was guided through a review of the literature and to determine a theoretical model for the chatbot’s basis: the habit formation model Conducted this formative usability test prior to the randomized controlled trials below to identify issues and make revisions	Literature review Prototype testing	Unable to assess
Piao et al [35], Canada	Randomized controlled trial	Text-based exercise chatbot	Literature review of extrinsic and intrinsic reward systems Steps for development were documented in the usability study described above	Literature review	Unable to assess
To et al [34], Canada	Nonrandomized experimental study	Text-based exercise chatbot	Development was outsourced for technical development by SmartAI. Did not report if the research team was involved in any other steps for development	None identified	Unable to assess

^a^GRIPP2: Guidance for Reporting Involvement of Patients and Public.

^b^PPI: patient and public involvement.

^c^SCD: sickle cell disease.

### Study Design and Interventions

Three of the included studies were RCTs [31,35,42], 4 were nonrandomized experimental studies [32,34,36,41], 3 were user-design and development studies [30,37,40], 3 were usability studies [38,39,43], 1 was a feasibility study [28], and 2 were proof-of-concept studies [29,33].

Fifteen of the 16 included studies reported the sample size; sample sizes ranged from 18 to 116 participants [34,37]. Participants’ age ranged from 12 to 69 years, with most participants being younger than 50 years old. When a specific chronic disease group was described, populations included patients with celiac disease [42], diabetes [28,32], cancer [31], and sickle cell disease [38]. Where reported, the inclusion of female participants ranged from 31.4% to 100% [37]. Five studies involved an exercise intervention [34,35,37,39,43]. Three studies included a mental wellness intervention for healthy coping, life skill coaching, and positive psychology skill building [29-31]. Two studies evaluated a nutrition intervention [41,42]. The remaining interventions combined exercise, nutrition, and mental wellness components [28,32,33,36,38,40]. Across all reviewed articles, the chatbots communicated with users through text.

### Study Findings

There were several approaches used to guide the development and training of chatbots. In 3 of the included studies, the nonsoftware development approaches for chatbot development were not documented; therefore, no approaches were identified [33,34,36]. Thirteen studies reported approaches taken for chatbot development, with most studies reporting multiple approaches [28-32,35,37-43]. In 4 of the 13 studies, patients were engaged as knowledge experts or participants in co-design workshops [29,30,37,42]. In 6 of the 13 studies, patients were involved in the study as research participants and, as part of the study outcomes, were invited to share their views through interviews, prototype testing, and the Wizard of Oz (WoZ) procedure [28,31,37,40,42,43]. Ten of these 13 studies used a literature review, an approach that did not involve patients [28,31,32,35,38-43]. Notably, 7 of the 16 included studies were already at a more advanced stage of chatbot development, focusing on evaluating interventions and usage instead of focusing on the development process itself [31,32,34-36,41,42]. Within these studies, researchers often briefly described their overall approaches but did not go into detailed steps or explain why those steps were considered important. This did range from study to study. In 1 nonrandomized experimental study, it was reported that development was outsourced to a software company without further details regarding the process [36]. In contrast, 1 RCT effectively described the formative work their team did working with patients to refine content through interviews and focus groups [31]. However, the degree of utilization and success of the development strategy was not discussed [31]. Although we searched the literature for formative papers that preceded the included papers, no additional studies were identified using this approach (Figure 1). These nontechnical development approaches are listed and described in more detail below.

### Collaboration With Patient and Clinician Partners as Knowledge Experts

During the early stages of chatbot planning, 2 studies consulted experts for chatbot development [29,42]. In both studies, patient partners were recognized as knowledge experts and included as part of the research team [29,42]. In the study with a nutrition chatbot for a celiac disease patient group, patients were recognized as experts alongside health care professionals, including dietitians and gastroenterologists [42]. In the mental wellness study, a team of 3 clinical psychologists took part in chatbot intervention development and content refinement alongside 2 users and a group of behavior change experts; this iterative process was used to adapt the chatbot’s intervention program, and audiovisual content to user needs through a clinical lens [29].

### Co-design Workshops

Two studies used co-design workshops to allow patients to creatively engage in the development of content ideas, chatbot design, chatbot style elements, and chatbot use [30,37]. One study invited participants to collaborate and develop ideas together with the research team over Zoom (a web-based communication platform; Zoom Video Communications, Inc) by visualizing ideas on Google Jamboard software (a web-based whiteboard for idea sharing) [37]. Another study invited patients to use a prototyped session with the chatbot to collect their needs, content preferences, stylistic ideas, and suggestions for improvements [30].

### Interviews With Patients

In 5 studies, patient interviews were conducted beforehand to guide chatbot development by exploring patient needs, perceptions, and experiences with chatbot use and healthy living [28,31,37,40,42]. In 1 study, interviews were administered during prototype testing and analyzed qualitatively [37]. Another study conducted this formative work through focus groups and interviews to collect information from young adults treated for cancer, the target end user population [31]. This information was then used to guide chatbot content development within a patient-centered lens. Follow-up interviews were conducted after interventions or chatbot exposure [28,40]. Questionnaires and surveys were also used in addition to interviews to collect similar information from patients [28,42].

### Prototype Testing

Many included studies were nonexperimental or pilot studies used to assess the feasibility and measure usability. These formative studies can be considered a step for development before releasing and testing a mature chatbot in an RCT. For example, 1 study using a chatbot for an exercise intervention organized a 3-week formative usability study [43] to identify issues and make revisions before conducting an RCT [35].

### WoZ Procedure

One study used the WoZ procedure [37] (where the technology is controlled by a human interface in chatbot development) as a step in their chatbot development. This procedure is administered by engaging participants in a 20-minute conversation with a simulated chatbot that was not automated but controlled manually by a researcher answering questions on the back end [37]. This step was developed to understand how the chatbot should interact with humans in a natural setting and to collect content-related information directly from participants [37].

### Use of Existing Literature to Gain Evidence-Based Knowledge for Development

In 10 studies, initial literature reviews were completed to gain evidence-based knowledge to guide chatbot development [28,31,32,35,38-43]. In 3 of these 10 studies, a literature review was used to develop content from evidence-based sources, including self-management practices, clinical guidelines, and systematic reviews [28,32,38]. A mental wellness study incorporated this step into development by reviewing the psychological theories and practices used to create the lessons the chatbot would deliver [31]. In another study, a literature review of the existing health behavior change models was conducted to understand the pros and cons of each model, and to guide the development of a novel behavior change model to structure the chatbot’s content [42]. In 1 study, gray literature was sourced through web-based chat threads for a youth helpline, so researchers could better understand content topic preferences and expected answers [40]. Finally, 2 of these 10 studies reviewed the literature to learn more about reward systems and to identify a theoretical basis for chatbot development [35,43].

### Patient Engagement and Public Involvement

Overall, the reporting of patient engagement in our included studies was limited making an assessment of PPI using the GRIPP2 challenging. Though 8 studies in our review reported involving patients, 5 provided inadequate detail, making assessing patient involvement impossible [28,29,31,42,43]. Specifically, these studies did not report on the aim of PPI, did not clearly articulate their methods, or did not discuss the role of PPI in their outcomes. The remaining 8 studies were not evaluated using the GRIPP2 because they did not report development approaches at all [33,34,36] or did not involve patients in the reported approaches [32,35,38,39,41].

Of the 3 studies we assessed using the GRIPP2, 1 study scored 3 points on the GRIPP2 Field [37], with the other 2 scoring 1 point [30,40]. Figueroa et al’s study scored 3/5 on the GRIPP2 scale [37]. This study provided a clear description of the methods used for PPI, commenting on how PPI influenced the study and on successful and unsuccessful aspects of the study relating to PPI [37]. This study was also the only one that described 4 different approaches used for development, including co-design workshops, interviews, WoZ, and prototype testing. The authors noted that their co-design sessions “brought unexpected participant preferences and wishes, which were useful in developing subsequent versions” of their chatbot [37]. Further, they recognized the importance of engaging patients in design, testing, and dissemination to develop chatbot interventions that participants would use and benefit from. The remaining 2 studies, 1 by Gabrielli et al [30] and the other by Maenhout et al [40], were each awarded a single point on the GRIPP2 for clearly describing the methods used for PPI. The reporting was such that future researchers could replicate similar development approaches to actively engage patients in research design.

## Discussion

### Principal Findings

In this review, we described the nontechnical approaches taken for chatbot development and evaluated the extent of patient engagement using the GRIPP2. While promising approaches were shared about the nontechnical steps associated with chatbot development, the level of detail provided was often low, including how patients were involved in the process.

The limited level of detail speaks to the need to prioritize frameworks for implementing digital health tools [44,45]. This will involve a focus on increased formative, development, and feasibility studies and a shift to implementation research that considers embedding and sustaining interventions in context [44,45]. A more detailed focus on the developmental stages and implementation process in research would allow increased replicability of developmental approaches that actively engage patients and progress the field of chatbot research from the end user perspective. An example of this focus on the implementation process includes the formative work conducted by Islam and Chaudhry [46] while developing a chatbot to support the health care needs of patients during the recent COVID-19 pandemic. Their work is an example of detailed documentation of a replicable multi-phased chatbot design study, offering guidance for future research in this area [46]. Additional focus on implementation will ensure the production and monitoring of chatbots that provide quality care and service to patients across short- and long-term timelines [44]. This strategic planning also holds promise to better respond to the requirements of diverse user cohorts, especially those with lower levels of digital health literacy [47].

Although an attempt was made to evaluate the extent of the patient engagement process by the GRIPP2 patient engagement checklist, due to limited detail of reporting, this was only possible in 3 studies [30,37,40]. Many digital health solutions are plagued with low uptake and poor usability as they were developed with minimal patient involvement [48]. As user-centered design and patient engagement are known to improve the quality of research, using engagement approaches throughout the research continuum could result in the identification of system requirements that would be otherwise missed, as well as result in a better understanding of patient needs, higher intervention engagement, and increased intervention effectiveness [49]. Some of the approaches we have identified in this review, including co-design workshops, the WoZ approach, patient interviews, and iterative prototype testing, represent ways researchers can creatively and actively engage patients throughout the development process. Co-design workshops foster a richer understanding of what patients “know, feel, and even dream” [50]. The WoZ approach is a widely accepted evaluation and prototyping methodology for developing human-computer interaction technology [51]. Engaging patients in iterative prototyping and user testing cycles has proven to improve the ease of use and adoption of these interventions [52]. In alignment with the literature, we recommend that researchers taking on health chatbot development projects consider adopting approaches such as co-design workshops, interviews, WoZ, and prototype testing.

**Figure 2:**
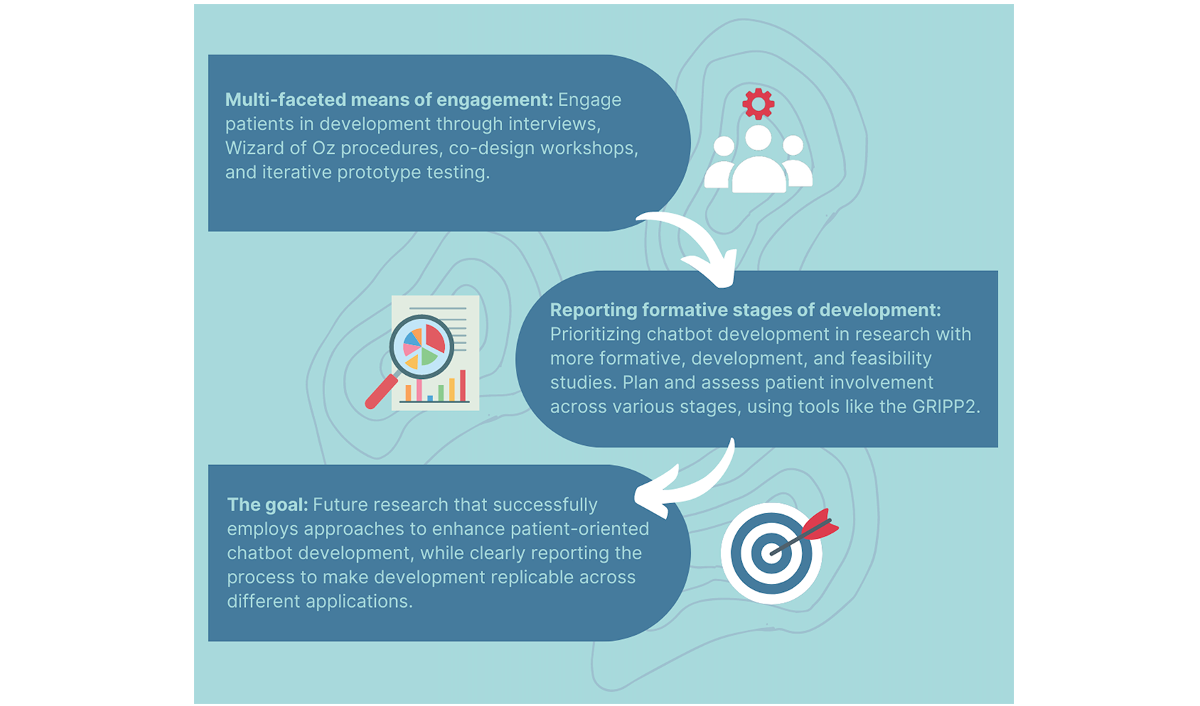
Informing areas of future research in patient-oriented chatbot development for lifestyle and wellness interventions. GRIPP2: Guidance for Reporting Involvement of Patients and Public.

Despite the available evidence supporting the benefit of patient involvement in intervention development, there are reasons why approaches that do not directly or actively involve patients may be more appealing to researchers. This notably includes challenges associated with recruitment, particularly when trying to avoid recruitment bias, and the time and resource intensity associated with the overall process [20]. The scarcity of patient involvement may also be related to an underappreciation of the potential benefits of patient involvement in digital health research and a limited understanding of how best to get patients involved [20]. Researchers and practitioners should be aware that there are many different approaches, strategies, and models to engaging patients in chatbot development. We have summarized some approaches in this review, and resources such as the Strategy for Patient-Oriented Research patient engagement framework and the patient engagement in research plan offer practical information to guide patient involvement in the development process [53,54]. Patients can participate at all stages, helping to define health care problems, identify solutions, participate as co-designers of an intervention, and refine the evaluation process [19]. Figure 2 offers the direction in informing future research in patient-oriented chatbot development for lifestyle and wellness interventions, including the application of multifaceted means of patient engagement, use, and thorough documentation of approaches to enhance chatbot development, and clear and replicable reporting of the formative stages of development.

### Strengths

We searched 9 of the most relevant bibliographic databases for medical and technology research for this review. No restrictions were placed on the year of publication, country of publication, journal, or study setting. Our study team consisted of multidisciplinary research and health care professionals with relevant expertise who provided direction at each review phase. This review was guided using an established framework proposed by Arksey and O’Malley [21].

### Limitations

This review focused on simple voice- or text-based chatbots that engaged in 2-way communication with human users. This led to the exclusion of other forms of conversational agent technology (ie, embodied conversation agents, humanoid and social robots, wearable technology, IoT, virtual avatars, interactive voice assistants, etc) that may have resulted in the finding of additional development and engagement approaches that were not covered in our review. Our review excluded literature from conference proceedings, protocol papers, and other papers lacking an intervention. Moreover, although our proportionate agreement was 0.967 at the title and abstract screening stage, there was only “fair” agreement between reviewers (Cohen κ=0.309). This “fair” agreement between researchers highlights the challenges in reviewing a heterogeneous body of literature. With ongoing meetings and refinement of our inclusion and exclusion criteria, the Cohen κ statistic improved to an “almost perfect” agreement at the full-text review stage (Cohen κ=0.843). Additionally, due to the limited detail available within the included studies, our team could not conclusively assess patient involvement in chatbot development; greater attention to reporting patient involvement in chatbot development and testing in future research will help with this limitation. Finally, we acknowledge that scoping reviews have numerous shortcomings, including limitations of rigor and potential bias stemming from the absence of a quality assessment, among others [55]. However, the literature on chatbot technology remains highly heterogeneous at this time, and scoping review provided a systematic method to map the current state of the literature.

### Conclusion

In conclusion, this review provides a menu of options that can be used for the nontechnical steps associated with chatbot development for interventions supporting lifestyle and wellness interventions. The identified study limitations hold promise to guide the inclusion of patient engagement and the improved documentation of the engagement and development of chatbots in future health care interventions. Given the importance of end user involvement in the development of digital technology, we hope that future research on chatbot development will take the opportunity to carry out a more systematic reporting of the chatbot development and implementation process and will actively engage patients as key members of the codevelopment process.

## Appendix

Multimedia Appendix 1 Search strategy documentation.

## Abbreviations

AI: artificial intelligence
DHI: digital health intervention
GRIPP2: Guidance for Reporting Involvement of Patients and Public
IoT: Internet of Things
PPI: patient and public involvement
PROSPERO: International Prospective Register of Systematic Reviews
RCT: randomized controlled trial
WoZ: Wizard of Oz
