# Supplementary material for: Examining Patient Engagement in Chatbot Development Approaches for Healthy Lifestyle and Mental Wellness Interventions: Scoping Review
Source: J Particip Med. 2023 May 22;15:e45772. doi: 10.2196/45772 (PMC10242458; doi:10.2196/45772)
Supplement: Multimedia Appendix 1 [file jopm_v15i1e45772_app1.pdf]

Supplementary Material – Search Strategy

Ovid MEDLINE(R) ALL 1946 to April 13, 2022, Embase 1974 to April 13, 2022

#	Search Statement	Results
1	(chatbot* or "im bot" or "im bots" or "instant message bot*" or "conversational agent*" or "virtual agent*").mp.	1615
2	remove duplicates from 1	1034

APA PsycInfo <1806 to July Week 2 2022>

#	Search Statement	Results
1	(chatbot* or "im bot" or "im bots" or "instant message bot*" or "conversational agent*" or "virtual agent*").mp.	774
2	*"Diets"/	10562
3	*"Health Promotion"/	22624
4	*"Intervention"/	52188
5	*"Physical Activity"/	19894
6	"Nutrition"/	12232
7	"Weight Loss"/	4330
8	"Sedentary Behavior"/	2138
9	(lifestyle* or health* or medic* or nursing or nurse* or disabilit* or elder* or "senior citizen*" or patient* or exercise or "physical activit*" or motivational).mp.	1881217
10	2 or 3 or 4 or 5 or 6 or 7 or 8 or 9	1905300
11	1 and 10	225

CINAHL Plus with Full Text
Search Modes: Find all my search terms

#	Query	Results
S1	(chatbot* or "im bot" or "im bots" or "instant message bot*" or "conversational agent*" or "virtual agent*")	429

Cochrane Library Searched July 08, 2022

ID	Search	Hits
#1	((chatbot*)):ti,ab,kw	106
#2	("im bot" or "im bots" or "instant message bot" or "conversational agent" or "virtual agent"):ti	20
#3	("im bot" or "im bots" or "instant message bot" or "conversational agent" or "virtual agent"):ab	62
#4	#1 or #2 or #3	160

IEEE Explore Searched July 13, 2022 Result =368

(((All Metadata:chatbot) OR (All Metadata:"im bot") OR (All Metadata:"instant message bot") OR (All Metadata:"virtual agent*") OR (All Metadata:"conversational agent*") AND ((All Metadata:lifestyle*) OR (All Metadata:medic*) OR (All Metadata:health*) OR (All Metadata:patient*) OR (All Metadata:nurs*) OR (All Metadata:elderly))

SCOPUS Searched July 13, 2022 Results=765

(TITLE (lifestyle* OR health* OR medic* OR nursing OR nurse* OR disabilit* OR elder* OR "senior citizen*" OR patient* OR exercise OR "physical activit*" OR motivational OR diet* OR nutrition* OR "weight loss*" OR "health promotion*")) AND (TITLE-ABS (chatbot* OR "im bot" OR "im bots" OR "instant message bot*" OR "conversational agent*" OR "virtual agent*"))

PROQUEST Dissertations & These Global Searched July 13, 2022 Results =65

noft(((lifestyle* OR health* OR medic* OR nursing OR nurse* OR disabilit* OR elder* OR "senior citizen*" OR patient* OR exercise OR "physical activit*" OR motivational OR diet* OR nutrition* OR "weight loss*" OR "health promotion*")) AND ((chatbot* OR "im bot" OR "im bots" OR "instant message bot*" OR "conversational agent*" OR "virtual agent*")))

PROSPERO Searched July 13, 2022 Results =40

(lifestyle* OR health* OR medic* OR nursing OR nurse* OR disabilit* OR elder* OR "senior citizen*" OR patient* OR exercise OR "physical activit*" OR motivational OR diet* OR nutrition* OR "weight loss*" OR "health promotion*") AND (chatbot* OR "im bot" OR "im bots" OR "instant message bot*" OR "conversational agent*" OR "virtual agent*")
